# Pharmacological Modulation of Neurite Outgrowth in Human Neural Progenitor Cells by Inhibiting Non-muscle Myosin II

**DOI:** 10.3389/fcell.2021.719636

**Published:** 2021-09-17

**Authors:** Julianna Lilienberg, Zoltán Hegyi, Eszter Szabó, Edit Hathy, András Málnási-Csizmadia, János M. Réthelyi, Ágota Apáti, László Homolya

**Affiliations:** ^1^Institute of Enzymology, Research Centre for Natural Sciences, Budapest, Hungary; ^2^Molecular Psychiatry and in vitro Disease Modelling Research Group, National Brain Research Project, Hungarian Academy of Sciences and Semmelweis University, Budapest, Hungary; ^3^MTA-ELTE Motor Pharmacology Research Group, Eötvös Loránd University, Budapest, Hungary; ^4^Motorpharma, Ltd., Budapest, Hungary; ^5^Department of Psychiatry and Psychotherapy, Semmelweis University, Budapest, Hungary

**Keywords:** human neural progenitor cells (hNPCs), blebbistatin, neurite, non-muscle myosin II, extracellular matrix (ECM)

## Abstract

Studies on neural development and neuronal regeneration after injury are mainly based on animal models. The establishment of pluripotent stem cell (PSC) technology, however, opened new perspectives for better understanding these processes in human models by providing unlimited cell source for hard-to-obtain human tissues. Here, we aimed at identifying the molecular factors that confine and modulate an early step of neural regeneration, the formation of neurites in human neural progenitor cells (NPCs). Enhanced green fluorescent protein (eGFP) was stably expressed in NPCs differentiated from human embryonic and induced PSC lines, and the neurite outgrowth was investigated under normal and injury-related conditions using a high-content screening system. We found that inhibitors of the non-muscle myosin II (NMII), blebbistatin and its novel, non-toxic derivatives, initiated extensive neurite outgrowth in human NPCs. The extracellular matrix components strongly influenced the rate of neurite formation but NMII inhibitors were able to override the inhibitory effect of a restrictive environment. Non-additive stimulatory effect on neurite generation was also detected by the inhibition of Rho-associated, coiled-coil-containing protein kinase 1 (ROCK1), the upstream regulator of NMII. In contrast, inhibition of c-Jun N-terminal kinases (JNKs) had only a negligible effect, suggesting that the ROCK1 signal is dominantly manifested by actomyosin activity. In addition to providing a reliable cell-based *in vitro* model for identifying intrinsic mechanisms and environmental factors responsible for impeded axonal regeneration in humans, our results demonstrate that NMII and ROCK1 are important pharmacological targets for the augmentation of neural regeneration at the progenitor level. These studies may open novel perspectives for development of more effective pharmacological treatments and cell therapies for various neurodegenerative disorders.

## Introduction

Neurodegenerative disease conditions, such as Huntington’s disease, Alzheimer’s disease, and Parkinson’s disease are characterized by a progressive loss of various types of neurons. Regenerative mechanisms, although limited in adult neural tissues, may delay or even halt disease progression. A major determinant of the reduced neuronal regeneration is the diminished axonal growth capacity of mature neurons. Extensive studies have been performed to elucidate the process of axonal growth impairment (Allingham et al., 2005; Medeiros et al., 2006; Tornieri et al., 2006; Rosner et al., 2007; Kubo et al., 2008; Kollins et al., 2009; Hur et al., 2011; Pool et al., 2011; Yu et al., 2012; Roland et al., 2014; Spedden et al., 2014; Xie et al., 2014; Evans et al., 2017; Wang et al., 2017, 2020; Tanaka et al., 2018; Basso et al., 2019; Dupraz et al., 2019; Costa et al., 2020), and, in addition to the intrinsic factors, revealed the importance of environmental inhibitory mechanisms in retarded axonal growth (Ichikawa et al., 2009; Hur et al., 2011; Tan et al., 2011; Beller and Snow, 2014; Baldwin and Giger, 2015; Nakamura et al., 2019). Because of the limited availability of human neural tissues, most of these studies used rodent or avian models.

Introduction of human pluripotent stem cells (PSCs) opens a new perspective for neurobiological research. Although application of stem cells obtained from early embryos raises several ethical issues, employing induced pluripotent stem cells (iPSCs) reprogrammed from adult tissues dispels these concerns. Differentiation of either embryonic stem cells (ESCs) or iPSCs into neural progenitor cells (NPCs), and subsequently into mature neurons, allows us to study human neural development and regeneration, as well as to explore the pharmacological modulation of neural cells of human origin.

Human neural progenitor cells, which represent *in vivo* a reservoir for neural regeneration, can be generated from human PSCs by directed differentiation. These cells resemble the usual cell lines in several aspects, as they can be passaged numerous times, cryopreserved, and transfected with various transgenes. At basal state, NPCs have a low number of short processes (1–2 per cell) with no substantial branching. However, when they start to differentiate into neurons, as an initial step, NPCs protrude projections, which subsequently elongate and branch. One of these projections differentiates into the extended axon, while the others become dendrites. Although the processes of NPCs greatly differ from those of mature neurons in both length and complexity, they can be considered as precursors of axons and dendrites. Therefore, we subsequently use the word “neurite” also for the projections of the neural progenitor cells.

Neurite outgrowth is a dynamic process; the elongation of these protrusions is not unidirectional but rather is a result of repeated growth and retraction. At the tip of these projections, actin dynamics has a crucial role. One of the factors governing actin dynamics is the non-muscle myosin II (NMII), which is an ATP-driven molecular motor protein responsible for the retrograde actin flow (recently reviewed in Costa and Sousa, 2020). Blebbistatin (BS) is a well-characterized, selective and potent inhibitor of NMII (Straight et al., 2003), and this compound blocks myosin heads in a low affinity state for actin, thus preventing formation of actomyosin complexes (Kovacs et al., 2004; Zhao and Overstreet-Wadiche, 2008). Despite that BS is widely used in studies on actomyosin network function, it holds a number of unfavorable properties, such as poor water-solubility, stability issues, fluorescence interference, cytotoxicity, and even phototoxicity (Roman et al., 2018). To overcome these negative properties of blebbistatin, several BS-derivatives have recently been developed. The most promising derivatives, which are non-fluorescent, highly soluble and non-toxic compounds, include para-nitroblebbistatin (NBS) and para-aminoblebbistatin (AmBS) (Kepiro et al., 2014; Varkuti et al., 2016; Rauscher et al., 2018; Gyimesi et al., 2021).

In the present work, we investigated the dynamics of neurite outgrowth in human stem cell-derived NPCs and examined the effect of BS and its derivatives on this process in permissive and inhibitory environments.

## Materials and Methods

### Generation of Pluripotent Stem Cell-Derived Neural Progenitor Cells Expressing Enhanced Green Fluorescent Protein

A human iPSC line, previously generated from fibroblasts of a healthy male individual by Sendai virus reprogramming (Vofely et al., 2018), was kindly provided by Fred H. Gage (Salk Institute), whereas the human ESC line, HUES9 was a kind gift from Douglas A. Melton (Howard Hughes Medical Institute). These cell lines were differentiated into NPCs by a directed differentiation protocol, which is based on a previously published, multistep procedure (Yu et al., 2014), somewhat modified in our laboratory (Supplementary Figure 1). Briefly, PSCs were cultured in mTeSR media (Stem Cell Technologies) on Matrigel-coated dishes (Corning), and passaged by ReLSR (Invitrogen). Embryoid bodies (EBs) were generated from human PSCs on low-adherence plates; then the cells were differentiated toward the neural lineage using DKK1 (PeproTech), SB431542 (Sigma), Noggin (Thermo Fisher Scientific), and cyclopamine (Sigma). After 21 days, the EBs were seeded onto plates previously coated with poly-ornithine/laminin (Sigma and Thermo Fisher Scientific), then cultured for an additional 7 days. Appearing rosettes were collected and dissociated, and then the cells were plated onto plates previously coated with poly-ornithine and laminin (see below). When attached NPCs became super-confluent, they were transferred onto new poly-ornithine/laminin-coated plates.

To obtain stable expression of enhanced green fluorescent protein (eGFP) in human NPCs, a Sleeping Beauty transposon-based gene delivery method was applied (Kolacsek et al., 2014). NPCs were co-transfected with a plasmid harboring a CAG promoter-driven eGFP and puromycin resistance gene (Supplementary Figure 2A), as well as with another vector containing CMV promoter-driven Sleeping Beauty transposase. Transfection was carried out using Fugene HD reagent (Thermo Fisher Scientific) according to the manufacturer’s instructions. Eight days following transfection, the cells were subjected to 1.6 μg/ml puromycin for 24 h. To obtain NPCs expressing eGFP at a high level (eGFP-NPCs), the cells were sorted by a FACS Aria cell sorter (Supplementary Figure 2B).

### Surface Coating and Cell Culturing

Six-well plates (Greiner) were coated with 2 ml of 10 μg/ml poly-ornithine solution for 24 h at room temperature (RT). The wells were then washed three times with phosphate-buffered saline (PBS; Thermo Fisher Scientific), and 2 ml of 5 μg/ml laminin solution was added for an additional 16 h at 4°C. A total of 3–5 × 10^5^ NPCs were seeded into each well, and cultured in DMEM/F-12, Glutamax medium supplemented with N2 Supplement-A, B27 (all from Thermo Fisher Scientific), basic fibroblast growth factor (Invitrogen), Antibiotic-Antimycotic (Gibco) and laminin (1 μg/ml). The culture medium was replaced every other day. Confluent wells were washed with PBS, and the cells were detached with a 5-min Accutase (Stem Cell Technologies) treatment.

For experiments, 96-well plates (Greiner) were coated with various extracellular matrices (ECMs) as follows: Matrigel, previously thawed on ice and dissolved in cold media (50 μg/ml), was distributed into pre-chilled 96-well plates (100 μl per well), which were then incubated for 24 h at 4°C. Alternatively, the plates were treated with 100 μl of poly-ornithine (10 μg/ml) or poly-lysine (100 μg/ml, Sigma) for 24 h at RT, followed by overlaying with 200 μl laminin (5 μg/ml), where indicated. Other surface treatments included coating with 100 μl of aggrecan (Aggr, 50 μg/ml, Sigma), chondroitin-sulfate (CS; 10 μg/ml, Merck), myelin oligodendrocyte glycoprotein (MOG, 50 μg/ml, Sigma), and chondroitin-sulfate proteoglycan (CSPG; 10 μg/ml, Merck) solutions for 24 h at 4°C.

### Neurite Outgrowth Measurements and Analysis

Two hours after seeding eGFP-NPCs onto 96-well plates previously coated with various ECM components (3–5 × 10^3^/well), the cells were subjected to NMII, Rho-associated, coiled-coil-containing protein kinase 1 (ROCK1), or c-Jun N-terminal kinase (JNK) inhibitors at various concentrations, as indicated. While NBS and AmBS were produced as described previously (Gyimesi et al., 2021), the ROCK1-selective inhibitor Y27632 and the pan-JNK inhibitor SP600125 were purchased from Sigma. Green fluorescence images were acquired by an ImageXpress Micro XLS instrument (Molecular Devices) equipped with environment control unit providing 37°C temperature and humidified atmosphere containing 5% CO_2_, 20% O_2_, and 75% N_2_. Six fields of view, covering approximately 40% of the total well surface (see Supplementary Figure 2C), were imaged for 4 h in 15 min intervals using an FITC filter cube (ex. 482/35 nm, em. 536/40 nm) and a 10× Nikon objective (Plan Fluor, NA = 0.3). All conditions were measured in three technical parallels.

For quantitative analysis, the total neurite lengths were assessed in each field of view using the Neurite Outgrowth module of MetaXpress software (Molecular Devices). The maximum width for cell bodies was defined as 150 μm not to exclude tight groups of cells from the analysis. The minimum fluorescence intensity of cell bodies was set to 3000 arbitrary units (AU) over the background on the 16-bit images (dynamic range 0–65,535). The criterion parameters for neurites were as follows: maximum width 10 μm, minimum fluorescence intensity 500 AU, and minimum extension from the cell body 20 μm (see Supplementary Figure 2D). For all analyses, the total neurite lengths were determined. To eliminate errors originating from initial cell number variations, the kinetic curves were normalized to initial cell numbers, and background was subtracted. To analyze the initial neurite growth rates, the first three points of the kinetic curves, covering a 30-min period, were linearly fitted, and the slope was used as an output parameter. For statistical analyses, non-parametric Kruskal–Wallis test was performed to compare a series of various experimental conditions, whereas Mann–Whitney tests were used to identify particular differences between individual sample pairs. The results are expressed as mean ± SEM obtained from at least three independent experiments.

## Results

### Stimulation of Neurite Outgrowth in Human Neural Progenitor Cells by Blebbistatin and Its Derivatives

**FIGURE 1:**
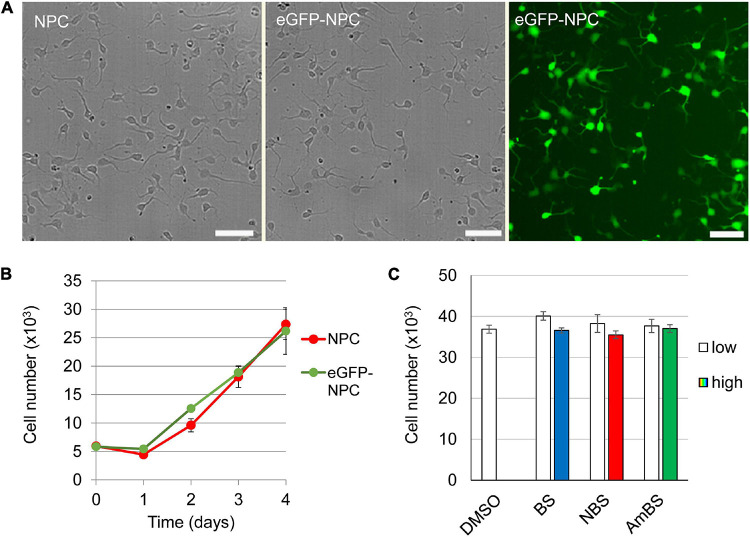
Characterization of human neural progenitor cells stably expressing eGFP. eGFF was stably expressed in human NPCs using a Sleeping Beauty transposon-based expression system. **(A)** No morphological difference between the parental (NPC) and the GFP-expressing NPCs (eGFP-NPC) can be observed. However, the transfectants exhibit uniform green fluorescence, making not only the cell body but also the neurites visible. **(B)** There is no difference in the proliferation capacity of NPCs and eGFP-NPCs. **(C)** The cytotoxic effects of blebbistatin (BS), para-nitroblebbistatin (NBS), and para-aminoblebbistatin (AmBS) was investigated by subjecting eGFP-NPCs (3 × 10^4^) to these compounds 2 h after seeding. BS and NBS were applied at 10 or 20 μM concentrations marked as low and high, respectively; whereas AmBS was used at 20 or 40 μM concentrations. The cell numbers were determined 4 h after the treatments and compared to the cell number of the vehicle (DMSO) treated sample. The mean ± SEM values from three independent experiments are shown in **(B,C)**. No significant differences were found.

**FIGURE 2:**
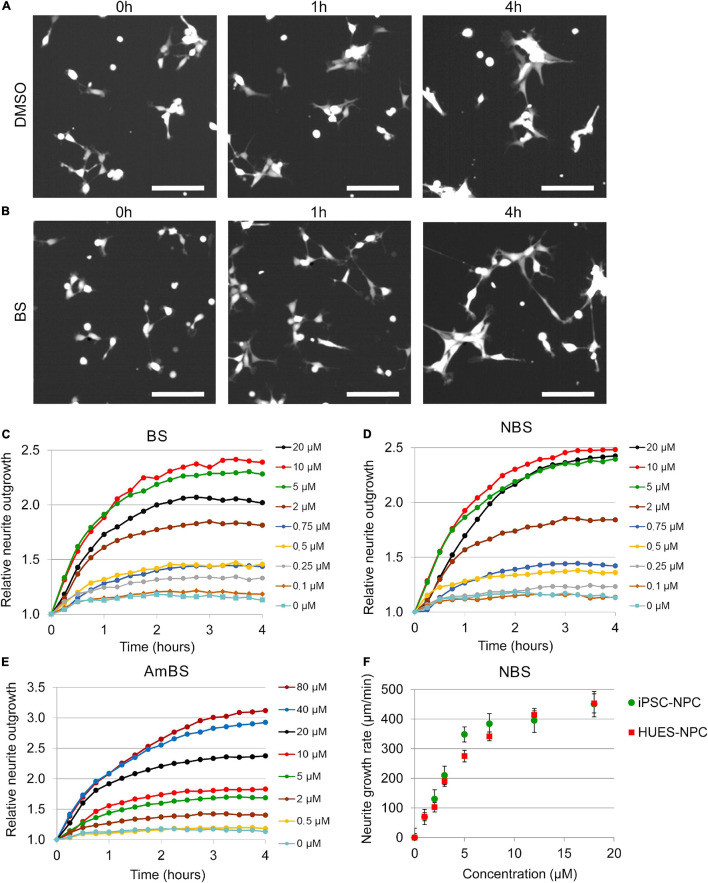
Effect of blebbistatin and blebbistatin derivatives on the neurite outgrowth of NPCs. **(A,B)** Representative images of NPCs stably expressing eGFP treated with vehicle (DMSO) or 10 μM blebbistatin (BS). Time points indicate elapsed time after treatment. **(C–E)** Kinetics of neurite growth in eGFP-NPCs subjected to BS, para-nitroblebbistatin (NBS), or para-aminoblebbistatin (AmBS) in the indicated concentrations. The total lengths of neurites were determined in six fields of view containing 120–150 cells in each condition. The neurite lengths were normalized to the initial values. All three compounds stimulated neurite outgrowth in a dose dependent manner. The EC_50_ values for BS and NBS were about 2 μM, whereas EC_50_ value for AmBS was above 10 μM. Panels **(C–E)** depict representative experiments. **(F)** Dose-response curves for NBS-stimulated neurite outgrowth analyzed in two human NPCs of different origin, i.e., iPSC-derived neural progenitor cells (iPSC-NPC) and NPCs differentiated from the human embryonic stem cell line HUES9 (HUES-NPC). Both NPC lines stably expressed eGFP. The neurite outgrowth rates were determined from the initial growth rates. The mean ± SEM values from three independent experiments are shown. No marked difference was observed between the two cell lines.

To study neurite dynamics in human neural progenitor cells, stem cell-derived NPCs expressing eGFP at high level were examined using a high-content screening and analysis system. Insertion of eGFP cDNA into the NPC genome caused no alteration in the cell morphology or the proliferative capacity (Figures 1A,B). High eGFP expression levels resulted in high-contrast images allowing visualization of even thin cellular projections (see Supplementary Figure 2D). Negligible phototoxic effect was observed even during long-term experiments with frequent illuminations (data not shown). As a poly-ornithine/laminin-coated surface was proposed as optimal condition for NPC culturing (Turney and Bridgman, 2005; Yu et al., 2014), the cells were first studied on this coating. Two hours after seeding the cells fully adhered to the surface, and started to protrude 1–3 projections per cell (2.1 ± 0.09 as an average). The steady state length of these neurites was approximately 20 μm (17.8 ± 0.3 μm) each with negligible branching (0.055 ± 0.008 branches per neurite) (Figure 2A). These steady state values were a result of recurrent elongation and retractions of the projections (Supplementary Video 1).

When NPCs were subjected to blebbistatin, rapid outgrowth of neurites was observed (Figure 2B and Supplementary Video 2). As shown in Figure 2C, BS stimulated neurite protrusion at a concentration as low as 0.25 μM, but its effect was more pronounced above 2 μM. The morphological changes observed in NPC treated with 20 μM BS may suggest toxic effect of BS at these higher concentrations, and can explain why less stimulatory effect was seen at 20 μM than at lower concentrations (5 and 10 μM). Less toxic and more water-soluble derivatives of BS, NBS, and AmBS also promoted neurite outgrowth in human NPCs (Figures 2D,E). Contrary to BS, these derivatives were fully effective even at high concentrations. It should also be noted that AmBS appeared to be less potent than BS and NBS. The maximal stimulatory effect of AmBS was achieved only at 40 μM concentration. The cytotoxicity of BS, NBS, and AmBS was also examined, but no significant reduction in the cell numbers were observed in the given time period (Figure 1C), although a slight decrease was seen, when BS or NBS was applied at higher concentration (20 μM).

The stimulatory effect of NBS on neurite outgrowth in NPCs differentiated from HUES9 cells was similar to that seen in iPSC-derived progenitor cells (Supplementary Figure 3). The initial rate of NBS-stimulated neurite outgrowth was determined in each kinetic curve, and dose-response relationships for both HUES- and iPSC-derived NPCs were generated. No substantial differences between these two types of NPC lines were observed (Figure 2F). The EC_50_ values for NBS stimulation in HUES- and iPSC-derived NPCs were 3.97 ± 0.34 and 3.11 ± 0.25 μM, respectively.

**FIGURE 3:**
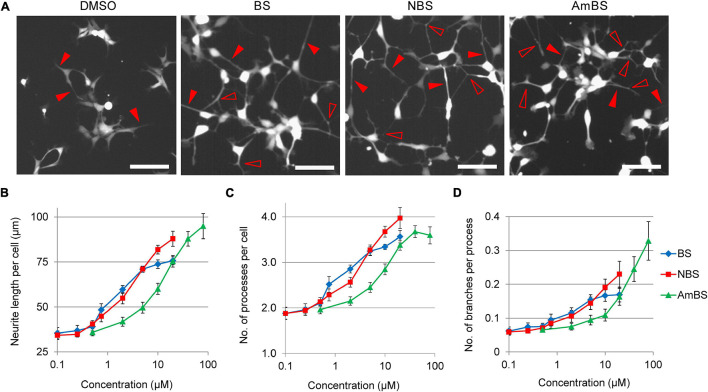
Morphological changes in NPCs treated with blebbistatin or blebbistatin derivatives. **(A)** Representative images of eGFP-NPCs treated with vehicle (DMSO), 20 μM blebbistatin (BS), 20 μM para-nitroblebbistatin (NBS), or 40 μM para-nitroblebbistatin (AmBS) for 4 h. Filled arrowheads point to selected processes, whereas empty arrowheads indicate branching points of the processes. Scale bars indicate 10 μm. **(B–D)** Dose response curves for neurite length per cell, the number of projections per cell, and neurite branching in response to BS, NBS, or AmBS. The values were determined 3 h after treatment. The mean ± SEM from three independent experiments are indicated.

In addition to the total neurite lengths in the studied fields of view, we analyzed cell morphology changes in response to BS compounds. At basal state, 2 h after seeding, NPCs possessed ∼2 neurites per cell with the total length of 30.0 ± 1.9 μm on average. After an additional 3-h period, the number of processes remained unaltered, but the length slowly elevated to 34.4 ± 1.9 μm per cell. However, when NPCs were treated with BS, NBS, or AmBS, the neurite lengths per cell extensively increased in a dose dependent manner reaching 73.8 ± 2.4, 87.9 ± 4.1, and 94.8 ± 7.0 μm per cell, respectively (Figures 3A,B). Interestingly, the number of processes also increased close to four neurites per cell (Figure 3C). In terms of neurite length, AmBS was the most efficacious, but less potent than the other two BS compound; whereas regarding number of processes, NBS was the most potent and most efficacious BS derivative. Under basal conditions, the neurites of NPCs exhibit hardly any branching: every 15th process has one branching. BS and BS derivatives stimulated neurite branching to some extent (Figure 3D). AmBS was the most effective in this regard: every third neurite showed branching after the treatment with 80 μM AmBS. It is noteworthy that the potency of AmBS for branching was even less than that for neurite length or process number.

### Effect of Extracellular Matrix Components on Neurite Outgrowth

**FIGURE 4:**
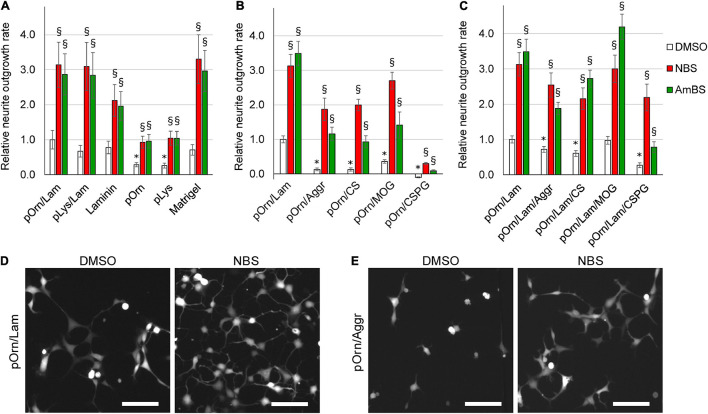
Effect of various extracellular matrices on the neurite outgrowth. **(A)** Human NPCs were seeded onto culturing plates coated with permissive ECM components, such as laminin, poly-ornithine (pOrn), poly-lysine (pLys), Matrigel, the combination of poly-ornithine and laminin (pOrn/Lam), or the combination of poly-lysine and laminin (pLys/Lam). Neurite outgrowth was monitored in the presence and absence of 10 μM para-nitroblebbistatin (NBS) or 20 μM para-aminoblebbistatin (AmBS). The neurite outgrowth rates were determined from the initial growth rates. The neurite outgrowth rate measured in cells seeded onto pOrn/Lam-coated surface was used as a reference point, and all growth rates were normalized to this value. **(B)** Neurite outgrowth rates were determined in NPCs plated onto coatings containing poly-ornithine (pOrn) in combination with restrictive ECM components, including aggrecan (Aggr), chondroitin-sulfate (CS), myelin oligodendrocyte glycoprotein (MOG), or chondroitin-sulfate proteoglycan (CSPG). As a reference point, the neurite outgrowth rate of cells on pOrn/Lam-coated surface was used. All four ECM components inhibited neurite outgrowth of NPCs. When the cells were treated with NBS or AmBS extensive neurite outgrowth was observed except for the cells seeded onto pOrn/CSPG-coated surface. **(C)** An experiment similar to that shown in **(B)** was performed with the difference that laminin was also included in the coatings. In this combination, MOG was not detrimental to the cells, and BS derivatives were able to override the inhibitory effect of not only Aggr and CS, but also of CSPG. In all panels, the mean ± SEM from at least three independent experiments are indicated. Asterisks indicate significant differences as compared to the samples grown on pOrn/Lam-coated surface, whereas section signs mark significant differences as compared to untreated samples (vehicle controls, white columns) (*p* < 0.05). **(D)** Morphology of NPCs seeded onto pOrn/Lam-coated surface and treated with vehicle (DMSO) or 20 μM NBS for 4 h. **(E)** Representative images of NPCs seeded onto pOrn/Aggr-coated plate and treated with DMSO or 20 μM NBS for 4 h. Scale bars indicate 10 μm.

To investigate how various ECM components influence neurite generation capability of human NPCs, the cells were seeded onto diverse ECMs. The frequently used, so-called growth-permissive extracellular matrices included Matrigel, laminin, poly-ornithine, poly-lysine, and combinations of poly-ornithine and laminin, as well as poly-lysine and laminin. Cell attachment and neurite outgrowth were substantially diminished, when human NPCs were seeded onto uncoated surface (data not shown). However, a steady basal neurite outgrowth was observed (2.51 ± 0.28 μm/min/cell 2 h after plating), when NPCs were plated onto poly-ornithine/laminin-coated surface. This condition was considered as a reference point for the experiments investigating the effect of various ECMs (Figures 4A–C). Similar neurite growth rates were seen in NPCs seeded on support coated with laminin, poly-lysine/laminin, or Matrigel, whereas poly-ornithine or poly-lysine alone provided suboptimal conditions for NPC neurite outgrowth (Figure 4A). It is important to note that these different ECMs markedly influenced cell morphology as demonstrated in Supplementary Figure 4. On poly-ornithine/laminin or Matrigel, the NPCs attached well, spread and formed small groups of cells with several outgrowths. NPCs seeded on poly-ornithine- or poly-lysine-coated support also adhered to the surface, but exhibited round-shape and remained dispersed. Despite these observations, the cell viability was not affected by these conditions (data not shown).

Next, we examined the effects of NBS and AmBS on the neurite formation of NPCs seeded on various ECMs. To achieve maximal stimulation and to avoid the toxic effects, NBS and AmBS were applied in 10 and 20 μM concentrations, respectively. We found that both blebbistatin derivatives significantly promoted neurite outgrowth (approximately threefold) regardless of the surface coating (Figures 4A,D). Since the basal neurite growth rates were smaller on poly-ornithine or poly-lysine, the stimulated ones were also less pronounced.

Several ECM components, mostly proteoglycans, produced primarily by oligodendrocytes in the vicinity of nervous system injury, were proposed as potential inhibitors of neuronal regeneration (Yu et al., 2012). Therefore, in the next set of experiments, we examined the effects of Aggr, CS, MOG, and CSPG on neurite outgrowth capacity of human NPCs. When laminin was replaced with these compounds in the extracellular coat, all studied ECM components strongly inhibited neurite generation (Figures 4B,E). Amongst them, CSPG was the most potent. When NPCs seeded on inhibitory coatings were subjected to NBS or AmBS at the maximum effective concentrations, these compounds were able to override the inhibitory effect of the ECM components. However, the stimulation of NBS did not reach the levels achieved with NPCs on the permissive coating (poly-ornithine/laminin). AmBS was even less effective; in most of the cases, its stimulation resulted in a neurite growth rate comparable with control level, i.e., NPCs on poly-ornithine/laminin without stimulation. However, the inhibition of CSPG was so profound that either NBS, or AmBS could promote neurite outgrowth to only a small extent.

Next, we investigated the effect of laminin, which is a key component of the basal membranes and provides efficient axon guidance. In a previous experiment (Figure 4A), we observed beneficial effect of laminin on NPC neurite outgrowth rate. When the extracellular coat contained laminin besides poly-ornithine and the inhibitory ECM components, the inhibitory effects of Aggr, CS, and CSPG were less pronounced but remained significant (Figure 4C). Moreover, the inhibitory effect of MOG was reversed. Here too, NBS and AmBS were able to override the effect of inhibitory ECM components. Their stimulatory effects were comparable to the ones found in NPCs on permissive coating with only one exception. AmBS was not that effective when employed to NPCs seeded onto CSPG-containing ECM, however, its stimulatory effect was still significant (Figure 4C).

### Role of Rho-Associated, Coiled-Coil-Containing Protein Kinase 1 and c-Jun N-Terminal Kinase in Neurite Outgrowth in Human Neural Progenitor Cells

**FIGURE 5:**
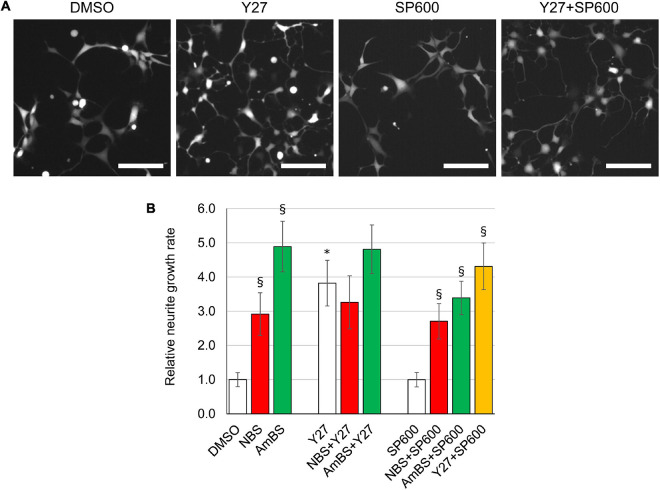
Effect of potential upstream regulators on basal and BS derivative-stimulated neurite outgrowth. **(A)** Representative images of eGFP-NPCs treated with vehicle (DMSO), 10 μM Y27632 (Y27, ROCK1 inhibitor), 10 μM SP600125 (SP600, JNK inhibitor), or with the combination of Y27 and SP600. Images were acquired 4 h after treatment. Scale bars indicate 10 μm. **(B)** The neurite outgrowth rates were determined in NPCs treated with NBS, AmBS, Y27, SP600, or combinations of those inhibitors. Vehicle control (DMSO) was used as a reference point. The ROCK1 inhibitor stimulated neurite outgrowth to similar extent to that seen with NBS and AmBS. In contrast, JNK inhibition had no effect on neurite outgrowth. When NBS was co-administered with Y27632 or SP600125, neither additivity nor inhibition was seen. Similar results were obtained with AmBS. Similar to NBS and AmBS, SP600125 did not alter Y27632-stimulated neurite outgrowth. All panels depict the means ± SEM from 3 to 5 independent experiments. Asterisks indicate significant differences as compared to the DMSO control, whereas section signs mark significant differences as compared to samples, which are not treated with NBS or AmBS in the same group (*p* < 0.05).

Next, we investigated two distinct signaling pathways, which have been reported to regulate neurite outgrowth in animal model systems. ROCK1 is an upstream regulator of NMII, and has been shown to increase actin-arc contraction and translocation rates (Zhang et al., 2003). JNKs have been reported to play a key role in the stabilization and bundling of microtubules with an enhanced activity during neurite generation (Tonges et al., 2011). NPCs seeded onto a poly-ornithine/laminin coated surface were subjected to inhibitors of ROCK1 or JNK, and the neurite outgrowth rate was assessed. The ROCK1-selective inhibitor, Y27632 significantly stimulated neurite outgrowth in NPCs to an extent comparable with that the blebbistatin derivatives, NBS and AmBS elicited (Figures 5A,B). In contrast, SP600125, a pan-JNK inhibitor had no effect on the neurite generation. The inhibitory potential of the compound was confirmed in an *in vitro* kinase assay (data not shown). The effect of the ROCK1 inhibitor was not additive when co-administered with either NBS or AmBS (Figure 5B). The JNK inhibitor had no marked effect on NBS- or Y27632-elicited neurite outgrowth, but slightly reduced the neurite growth rate when the NPCs were stimulated with AmBS. Our observations suggest that NMII and ROCK1 inhibition affect the same pathway modulating neurite outgrowth in human NPCs.

**FIGURE 6:**
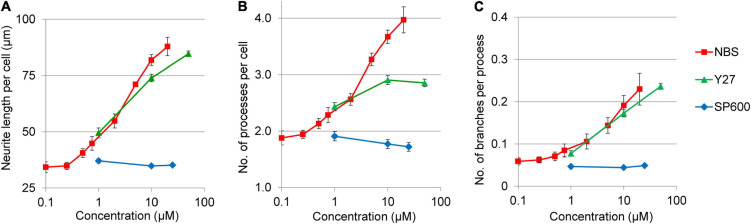
Dose response curves for various morphological parameters of NPCs treated with ROCK1 and JNK inhibitors. The neurite length per cell **(A)**, the number of projections per cell **(B)**, as well as the neurite branching **(C)** was stimulated by Y27632 (Y27) in dose dependent manners, whereas SP600125 (SP600) had no such effects. The values were determined 3 h after treatment. The NBS stimulated dose response curves were used as references. The mean ± SEM from three independent experiments are shown.

In addition to neurite growth rates, the morphology changes in response to Y27632 and SP600125 have also been analyzed (Figure 6). The ROCK1 inhibitor stimulated not only neurite length per cell, but also increased the number of processes per cell and the number of branches per neurites. In contrast, JNK inhibitor had no effect on any of these morphological parameters. Interestingly, the potency and efficacy of Y27632 was similar to NBS in terms of neurite growth and branching (Figures 6A,C), but not for the stimulation of the number of processes per cell, which plateaued at around three processes per cell (Figure 6B).

## Discussion

Neural cells, especially neurons, exhibit a unique polar architecture. Polarization of neural cells is an essential part of neuronal differentiation, thus being fundamental in neural network formation and nerve regeneration. One of the very first episodes of neural polarization is the protrusion of a number of processes from NPCs, followed by the elongation and branching of the evolving neurites. Eventually, one of the projections differentiates into the axon, whereas others develop into dendrites. A fine balance between positive and negative regulatory mechanisms, including both internal and environmental factors, ultimately leads to the establishment and maintenance of the neuronal structure. Development of cell protrusions is a complex dynamic process, the result of numerous events that involve not only membrane dynamics but also rearrangement of cytoskeletal elements. The structure of neurites is established by stable microtubule bundles and a highly dynamic mesh of filamentous actin (F-actin). Critical steps of neurite development include the elongation of microtubules at the positive ends, their bundling, and the continuous remodeling of actin filaments (Lowery and Van Vactor, 2009). The ATP-driven molecular motor protein, the non-muscle myosin binds to F-actin and controls the dynamics of actomyosin locomotion, a fundamental biological process, connected to numerous cellular and physiological functions including muscle contraction, cell differentiation, migration and polarization (Eggert et al., 2006; Vicente-Manzanares et al., 2009).

Blebbistatin is a widely used, potent inhibitor of NMII (Straight et al., 2003; Kovacs et al., 2004), and has been demonstrated as a useful tool for studying neuronal differentiation. Treatment of neural cells with blebbistatin results in extensive outgrowth of neurites as a consequence of abolished retrograde actin flow, when the balance of extension and retraction of cell protrusion is shifted to extension. Several studies employed blebbistatin to demonstrate the role of actomyosin contractility using primary neurons from mice (Kollins et al., 2009; Hur et al., 2011; Yu et al., 2012; Dupraz et al., 2019; Costa et al., 2020), rats (Allingham et al., 2005; Kubo et al., 2008; Kollins et al., 2009; Pool et al., 2011; Roland et al., 2014; Spedden et al., 2014; Evans et al., 2017; Wang et al., 2017, 2020; Tanaka et al., 2018; Basso et al., 2019; Costa et al., 2020), chickens (Rosner et al., 2007; Kollins et al., 2009; Xie et al., 2014), or even gastropods, such as *Aplysia* (Medeiros et al., 2006) and *Helisoma trivolvis* (Tornieri et al., 2006). These studies were performed in non-human primary neural cells, and investigated the neurite growth in matured neurons. The role of actomyosin in cannabinoid-induced changes in neuronal morphology was also established by blebbistatin inhibition in primary rat neurons and in Neuro2a murine neuroblastoma cells (Roland et al., 2014).

Few studies employed human PSC-derived neural models to investigate the involvement of actomyosin contractility in neural polarization and differentiation. Human ESCs were treated with blebbistatin to demonstrate the role of NMII in topography-induced neuronal maturation (Ankam et al., 2015). Blebbistatin antagonized differentiation of human iPSCs into midbrain dopaminergic neurons induced by mRNAs coding for proneural transcription factors, also implying the involvement of NMII (Xue et al., 2019). A large-scale screening for bioactive small molecules regulating neurite growth identified blebbistatin as a hit compound (108 hits out of 4421) (Sherman and Bang, 2018). For the high-throughput screen, iCell neurons were used, which are human iPSC-derived, cortical-like neural cultures consisting mostly of GABAergic interneurons. Contrary to the studies above, in which differentiated neurons were investigated, we examined the role of NMII in human neural progenitor cells, a cell population, which plays a pivotal role in the development of nervous system during ontogeny, as well as in neural regeneration. The NPCs applied in our study were committed to the hippocampal dentate gyrus lineage (Yu et al., 2014; Vofely et al., 2018). As dentate gyrus neural progenitor cells are essential for learning, pattern separation, and spatial memory formation, deviations in these cells can cause several disease conditions (Zhao and Overstreet-Wadiche, 2008). Thus, the investigation of neurite polarization mechanism in this cell type can help to improve strategies for neuro-regeneration and cell-based therapies. We found that blebbistatin induces not only elongation of existing neurites but stimulates generation of new projections and branch formation of neurites in human NPCs.

Although blebbistatin is widely used, it has numerous drawbacks when employed in cell biology applications. These include chemical instability, low solubility in water, toxicity to cells, and blue light-induced phototoxicity (Rauscher et al., 2018; Roman et al., 2018). Blebbistatin also tends to form fluorescent precipitates, which interfere with many cell biology assessments. A-ring modification of blebbistatin results in higher water solubility, but comes at the cost of lower potency for NMII inhibition (Verhasselt et al., 2017b). D-ring-modified blebbistatin analogs, however, such as 3′-hydroxy-blebbistatin, 3′-aminoblebbistatin (Verhasselt et al., 2017a), NBS (Kepiro et al., 2014), and AmBS (Varkuti et al., 2016), have more favorable properties, like higher water solubility, diminished cytotoxicity, and preserved potency. D-ring-modified BS derivatives with the exception of 3′-hydroxyblebbistatin are non-fluorescent at the spectral range normally used for microscopy or flow cytometry. In the present study, we demonstrated that NBS effectively stimulated not only neurite growth but also new protrusion generation and neurite branching with a potency similar to that of BS, whereas AmBS was less potent but more efficacious than BS in terms of neurite growth and branching stimulation.

Non-muscle myosin II is regulated by the phosphorylation of its regulatory light chain on Ser19 and Thr18, which is carried out by a number of kinases, including myosin light chain kinase, Rho-associated, coiled coil-containing kinase, leucine-zipper-interacting protein kinase, citron kinase, Serine/Threonine-protein kinase 21, and myotonic dystrophy kinase-related CDC42-binding kinase (Costa and Sousa, 2020). An equally important part of NMII regulation is its dephosphorylation by phosphatases, such as the myosin light chain phosphatase. The precise cellular localization of these kinases and phosphatases is crucial for the site-specific regulation of NMII, which is necessary to control filopodia dynamics. ROCK is a key component of various converging signaling pathways of upstream Rho-like GTPases. The regulatory role of MLCK and ROCK in neurite outgrowth has been demonstrated by numerous studies based on animal primary neural cell models (Tornieri et al., 2006; Rosner et al., 2007; Kubo et al., 2008; Kollins et al., 2009; Al-Ali et al., 2015; Wang et al., 2017). A recent report, using wild type and RhoA knockout mice as well as primary hippocampal neuron cultures from these animals, suggested a novel mechanism for Rho/ROCK signaling control of the axonal growth, i.e., ROCK restrains protrusion of microtubules to the leading edge of the growth cone by activating NMII-mediated actin arc formation (Dupraz et al., 2019). In concert with the animal-based neural models, we found that ROCK1 inhibition also promotes neurite outgrowth in human NPCs in a non-additive manner, indicating that ROCK1 is an upstream regulator of NMII also in this human neural model. These results also suggest that ROCK1 signal is manifested mainly by the NMII activity in the regulation of neurite outgrowth. It is worth noting again that previous studies applying animal-based systems investigated the role of NMII in mature neurons, focusing mainly on the growth cone or the axon initial segment, whereas in the present study, we aim at investigating a precursory event, the initialization of neurite formation in neural progenitors.

The JNK signaling pathway is two-faced in neural cells by means of promoting either cell development/regeneration or neuronal death/degeneration depending on the cell type, subcellular localization and cellular condition. A JNK1 knockout animal model demonstrated a pivotal role for JNK1 in neuronal microtubule assembly and stabilization (Chang et al., 2003). Subsequent studies using spiral ganglion or midbrain dopaminergic neurons from rats confirmed the involvement of all three JNK isoforms, although their differential contributions have also been revealed (Atkinson et al., 2011; Tonges et al., 2011). JNK3 was found to be the most prominent in mediating neurite regeneration and cell survival (Tonges et al., 2011). JNK phosphorylation of downstream effectors, such as the dendrite-specific high-molecular-weight microtubule-associated protein 2 (MAP2) and the microtubule-destabilizing protein superior cervical ganglion 10 (SCG10), were shown to contribute to defining dendritic architecture and axodendritic length, respectively (Bjorkblom et al., 2005; Tararuk et al., 2006). Contrary to these studies above, which were performed on mature neurons isolated from rodents, Lu et al. (2017) employed mouse embryonic neural stem cells (analogous to our NPCs) to examine the involvement of JNK. Inhibition of JNK diminished valproic acid-induced neurite outgrowth and neuronal differentiation in these cells. In our hands, the specific JNK inhibitor SP600125 did not block either basal neurite outgrowth, initiation of processes, or branching in human NPCs. Similarly, blocking the JNK pathway did not affect neurite growth elicited by either NMII or ROCK1 inhibition. Several reasons can be accounted for the different effect of JNK inhibition observed in the previously described cellular models and in our system. Interspecies difference between rodents and humans is one of the plausible explanations. Also, it is known that signaling events greatly dependent on the cell type. NPCs represent a tissue-specific stem cell population, in which regulatory mechanism can be different from that seen in mature neurons. In addition, previous studied revealed that specific cellular localization of kinases in either the RhoA- or the JNK-dependent pathway is crucial for the particular cellular functions (Coffey, 2014; Costa and Sousa, 2020). It is also noteworthy that we focused only on the initialization, the first 6 h of neurite generation. Kinetic differences can also be accounted for the conflicting results. Activation of JNK is relatively slow and its kinetics is site-specific in polarized neurons (Atkinson et al., 2011).

Restricted regeneration capability of the central nervous system (CNS) is determined by both intrinsic and environmental factors. The role of ECM in neural development and regeneration has long been studied. ECM components in the CNS are produced and secreted by both neurons and glial cells. Some of them, such as laminin and fibronectin, promote neural cell growth and migration, especially in the developing CNS, while others, e.g., CSPGs, serve as a barrier and prevent axons from growing into improper regions (Baldwin and Giger, 2015). Remodeled ECM at the site of the nervous system injury constitutes a detrimental environment, imposing a major obstacle for axonal regeneration (Beller and Snow, 2014). The effect of these permissive and restrictive ECM components on the neurite outgrowth has been demonstrated *in vitro* using matured neurons (Ichikawa et al., 2009; Hur et al., 2011; Tan et al., 2011; Nakamura et al., 2019); however, their impact on neural progenitor cells is poorly studied. In the present study, we showed that laminin was essential for maximal neurite growth capacity in human NPCs, and addition of laminin to the inhibitory ECM components weakened their detrimental effect. Moreover, BS derivatives were shown to override the ECM inhibition.

Cell therapies using stem cells or stem cell-derived transplants represent a promising and developing field of regenerative medicine. Several stem cell-based preclinical studies and also a limited number of clinical studies have been performed in connection with various CNS pathologies including neurodegenerative disorders (recently reviewed in Ford et al., 2020). Rodent models of Alzheimer’s disease and Huntington’s disease showed marked improvement in behavioral and cognitive deficits after transplantation of human iPSC-derived NPCs (Fujiwara et al., 2013; Jeon et al., 2014). Similarly, engraftment of human stem cell-derived NPCs or dopaminergic precursors ameliorated bradykinesia and drug-induced rotation behavior in various animal models of Parkinson’s disease (Roy et al., 2006; Kriks et al., 2011; Han et al., 2015). Moreover, early clinical trials have been launched or yet been forthcoming to explore the safety and efficacy of human iPSC-derived progenitors in Parkinson’s disease patients (reviewed in Parmar et al., 2020). Functional recovery was also demonstrated in stroke-damaged rodents subjected to human iPSC-derived NPC transplantation (Gomi et al., 2012; Mohamad et al., 2013). The key issue of these interventions is the functional integration of the transplanted cells. Neuronal polarization starting with protrusions of neurites is a prerequisite for NPCs to integrate. However, innate mechanisms and non-permissive nature of CNS environment for neurite growth greatly impedes this process. Our results demonstrate that targeting NMII can surmount both internal and environmental hindrance of neurite development, offering a new opportunity for improving effectiveness of integration of transplanted cells. Our *in vitro* data can serve as a base for future *in vivo* experiments to explore the potential of pharmacological augmentation of cell therapies for various CNS pathologies.

## Conclusion

In conclusion, we developed an *in vitro* experiment tool for quantitative assessment of neurite outgrowth in human hippocampal dentate gyrus neural progenitors. Using this system, we established the role of NMII in neurite generation, as well as its upstream regulation by the RhoA/ROCK1 signaling pathway. These results may provide new perspectives in the development of stem cells therapies as NMII might be considered as a novel drug target for integrating transplanted cells in neurodegenerative disorders, such as Parkinson’s and Alzheimer’s diseases, as well as in traumatic brain and spinal cord injuries (Ford et al., 2020; Lengel et al., 2020; Liu et al., 2020; Parmar et al., 2020; Bagheri-Mohammadi, 2021; Hu et al., 2021).

## Data Availability Statement

The original contributions presented in the study are included in the article/Supplementary Material, further inquiries can be directed to the corresponding author/s.

## Author Contributions

JL and ZH performed the experiments, analyzed the data, and prepared the original draft. ES and EH performed the experiments to generate NPCs stably expressing eGFP. AM-C, JR, and ÁA contributed to conceptualization and design of the study, and reviewing and editing of the manuscript. LH contributed to study conceptualization and design, supervision of experiments, data analysis, and writing and editing of the manuscript. All authors contributed to the article and approved the submitted version.

## Conflict of Interest

AM-C is an owner of Motorpharma, Ltd. Motorpharma, Ltd., has a license agreement with Eötvös Loránd University about the development and distribution of para-nitroblebbistatin. The remaining authors declare that the research was conducted in the absence of any commercial or financial relationships that could be construed as a potential conflict of interest.

## Publisher’s Note

All claims expressed in this article are solely those of the authors and do not necessarily represent those of their affiliated organizations, or those of the publisher, the editors and the reviewers. Any product that may be evaluated in this article, or claim that may be made by its manufacturer, is not guaranteed or endorsed by the publisher.

## Supplementary Material

Supplementary Figure 1 Generation of GFP-expressing NPCs. This scheme summarizes the multistage procedure how human neural progenitor cells stably expressing eGFP were generated from pluripotent stem cells. A human induced pluripotent cell line (iPSC) and a human embryonic stem cell line (HUES9) were seeded onto low-adherence plates to form embryoid bodies, which were subjected to DKK1, SB431542, Noggin, and cyclopamine for 21 days. Embryoid bodies were transferred to poly-ornithine/laminin-coated plates and cultured for an additional 7 days. Appearing rosettes were isolated, dissociated, and seeded again onto poly-ornithine/laminin-coated plates. In the stabilizing neural progenitor cells, eGFP was expressed using a Sleeping Beauty transposon-based gene delivery system (see Supplementary Figure 2). After selection with puromycin, NPCs expressing eGFP at high levels were sorted out by FACS.

Supplementary Figure 2 Assessing neurite outgrowth in NPCs expressing eGFP at a high level. **(A)** CAG promoter-driven eGFP sequence was inserted into Sleeping Beauty transposon vector, which also contained a puromycin resistance gene. The NPCs differentiated from pluripotent stem cells were co-transfected with this plasmid and another one harboring the sequence of the active transposase, resulting in insertion of the transgene into the genome of the NPCs. **(B)** After puromycin selection, the cells exhibited inhomogeneous eGFP expression levels (left panel). This cell culture was sorted for low and high (right panel) GFP-expressing populations. **(C)** GFP-expressing NPCs, seeded onto 96-well plates, were treated with various compounds. Six fields of view of each well were imaged in the indicated configuration. Image acquisition was performed for 4 h in 15-min intervals using a high-content screening equipment (ImageXpress Micro XLS, Molecular Devices). **(D)** During subsequent image analysis, on the basis of eGFP fluorescence and morphological parameters, the somas and neurites were identified at each timepoint using the Neurite Outgrowth module of MetaXpress software. Scale bars indicate 10 μm.

Supplementary Figure 3 Effect of para-nitroblebbistatin on neurite outgrowth in hESC-derived NPCs. An experiment similar to that shown in Figure 2D with iPSC-derived NPCs was performed with NPCs differentiated from the human embryonic stem cell line, HUES9. The NPCs were subjected to para-nitroblebbistatin in the indicated concentrations, and the total lengths of neurites were determined in six fields of view. The neurite lengths were normalized to the initial values. Initial neurite outgrowth rates were calculated from experiments similar to that depicted here, and a dose-response curve was generated (see Figure 2F).

Supplementary Figure 4 Cell morphology of NPCs grown on different extracellular matrices. eGFP-expressing NPCs were seeded onto various extracellular matrices as indicated (pOrn, poly-ornithine; pLys, poly-lysine). Images were acquired before and 4 h after the addition of para-nitroblebbistatin (10 μM). Scale bars indicate 10 μm.

Supplementary Videos 1, 2 Time-lapse images of NPCs in the presence and absence of NBS. NPCs expressing eGFP were subjected to DMSO (vehicle control, Supplementary Video 1) or 10 μM para-nitroblebbistatin (Supplementary Video 2), and the morphological changes were monitored for 4 h in 15 min intervals.

## Abbreviations

Aggr: aggrecan
AmBS: para-aminoblebbistatin
AU: arbitrary unit
BS: blebbistatin
CNS: central nervous system
CS: chondroitin-sulfate
CSPG: chondroitin-sulfate proteoglycan
EB: embryoid body
ECM: extracellular matrix
eGFP: enhanced green fluorescent protein
eGFP-NPC: enhanced green fluorescent protein-expressing neural progenitor cell
ESC: embryonic stem cell
HUES-NPC: human embryonic stem cell-derived neural progenitor cells
iPSC: induced pluripotent stem cell
iPSC-NPC: induced pluripotent stem cell-derived neural progenitor cells
JNK: c-JunN-terminal kinase
MAP2: microtubule-associated protein 2
MOG: myelin oligodendrocyte glycoprotein
NBS: para-nitroblebbistatin
NMII: non-muscle myosin II
NPC: neural progenitor cell
PBS: phosphate-buffered saline
PSC: pluripotent stem cell
ROCK1: Rho-associated, coiled-coil-containing protein kinase 1
RT: room temperature
SCG10: superior cervical ganglion 10.
