# Supplementary figures and images for: Pharmacological Modulation of Neurite Outgrowth in Human Neural Progenitor Cells by Inhibiting Non-muscle Myosin II
Source: Front Cell Dev Biol. 2021 Sep 17;9:719636. doi: 10.3389/fcell.2021.719636 (PMC8484915; doi:10.3389/fcell.2021.719636)

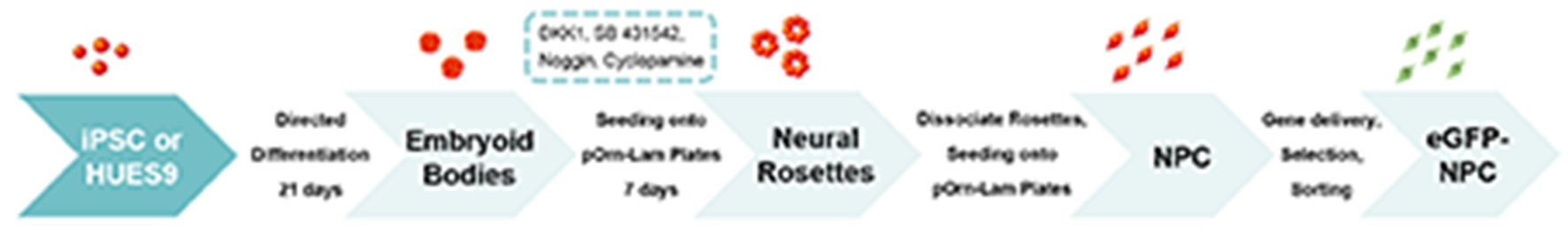

Supplement: Supplementary Figure 1 — Generation of GFP-expressing NPCs. This scheme summarizes the multistage procedure how human neural progenitor cells stably expressing eGFP were generated from pluripotent stem cells. A human induced pluripotent cell line (iPSC) and a human embryonic stem cell line (HUES9) were seeded onto low-adherence plates to form embryoid bodies, which were subjected to DKK1, SB431542, Noggin, and cyclopamine for 21 days. Embryoid bodies were transferred to poly-ornithine/laminin-coated plates and cultured for an additional 7 days. Appearing rosettes were isolated, dissociated, and seeded again onto poly-ornithine/laminin-coated plates. In the stabilizing neural progenitor cells, eGFP was expressed using a Sleeping Beauty transposon-based gene delivery system (see Supplementary Figure 2). After selection with puromycin, NPCs expressing eGFP at high levels were sorted out by FACS. [file Image_1.TIF]

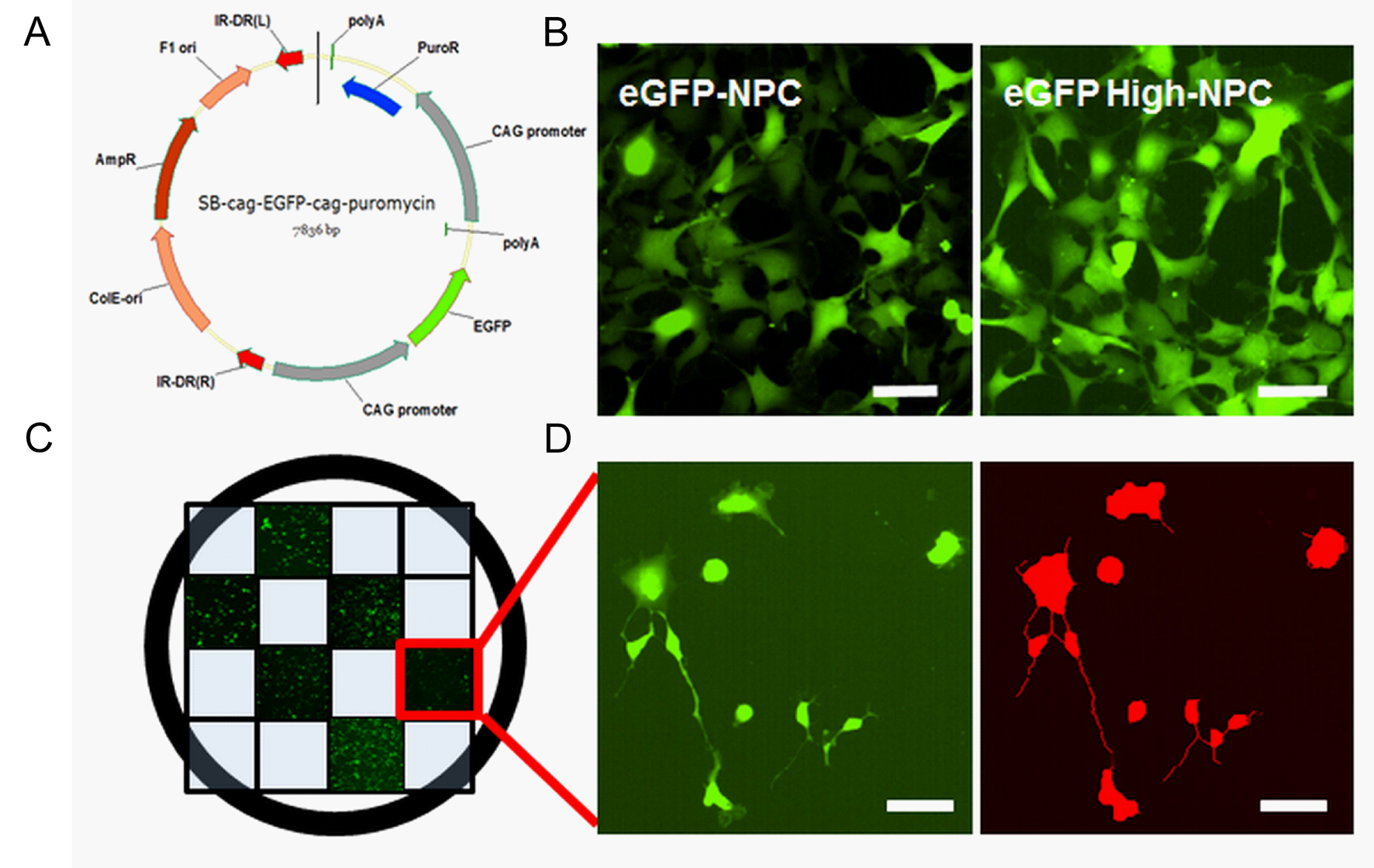

Supplement: Supplementary Figure 2 — Assessing neurite outgrowth in NPCs expressing eGFP at a high level. (A) CAG promoter-driven eGFP sequence was inserted into Sleeping Beauty transposon vector, which also contained a puromycin resistance gene. The NPCs differentiated from pluripotent stem cells were co-transfected with this plasmid and another one harboring the sequence of the active transposase, resulting in insertion of the transgene into the genome of the NPCs. (B) After puromycin selection, the cells exhibited inhomogeneous eGFP expression levels (left panel). This cell culture was sorted for low and high (right panel) GFP-expressing populations. (C) GFP-expressing NPCs, seeded onto 96-well plates, were treated with various compounds. Six fields of view of each well were imaged in the indicated configuration. Image acquisition was performed for 4 h in 15-min intervals using a high-content screening equipment (ImageXpress Micro XLS, Molecular Devices). (D) During subsequent image analysis, on the basis of eGFP fluorescence and morphological parameters, the somas and neurites were identified at each timepoint using the Neurite Outgrowth module of MetaXpress software. Scale bars indicate 10 μm. [file Image_2.TIF]

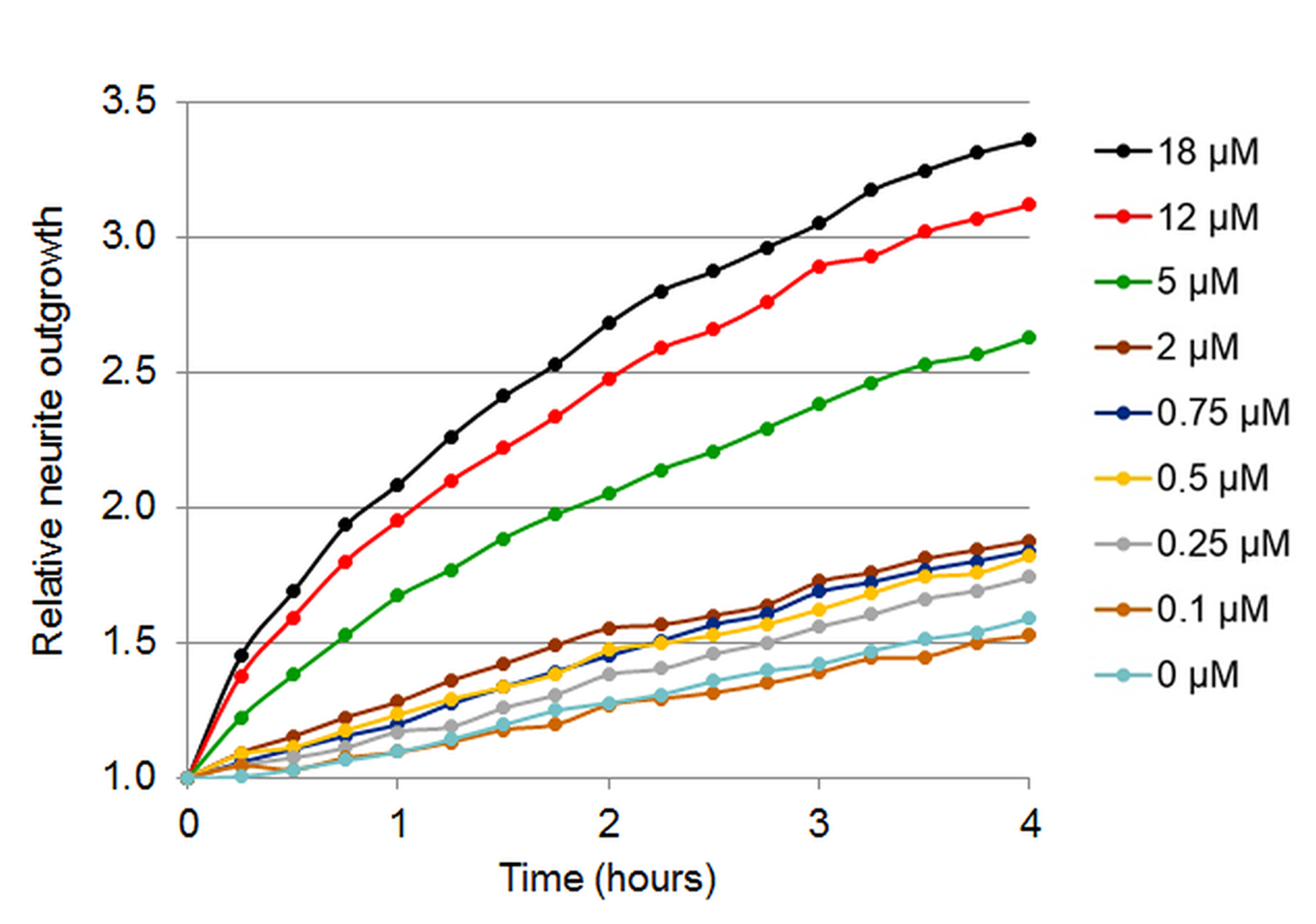

Supplement: Supplementary Figure 3 — Effect of para-nitroblebbistatin on neurite outgrowth in hESC-derived NPCs. An experiment similar to that shown in Figure 2D with iPSC-derived NPCs was performed with NPCs differentiated from the human embryonic stem cell line, HUES9. The NPCs were subjected to para-nitroblebbistatin in the indicated concentrations, and the total lengths of neurites were determined in six fields of view. The neurite lengths were normalized to the initial values. Initial neurite outgrowth rates were calculated from experiments similar to that depicted here, and a dose-response curve was generated (see Figure 2F). [file Image_3.TIF]

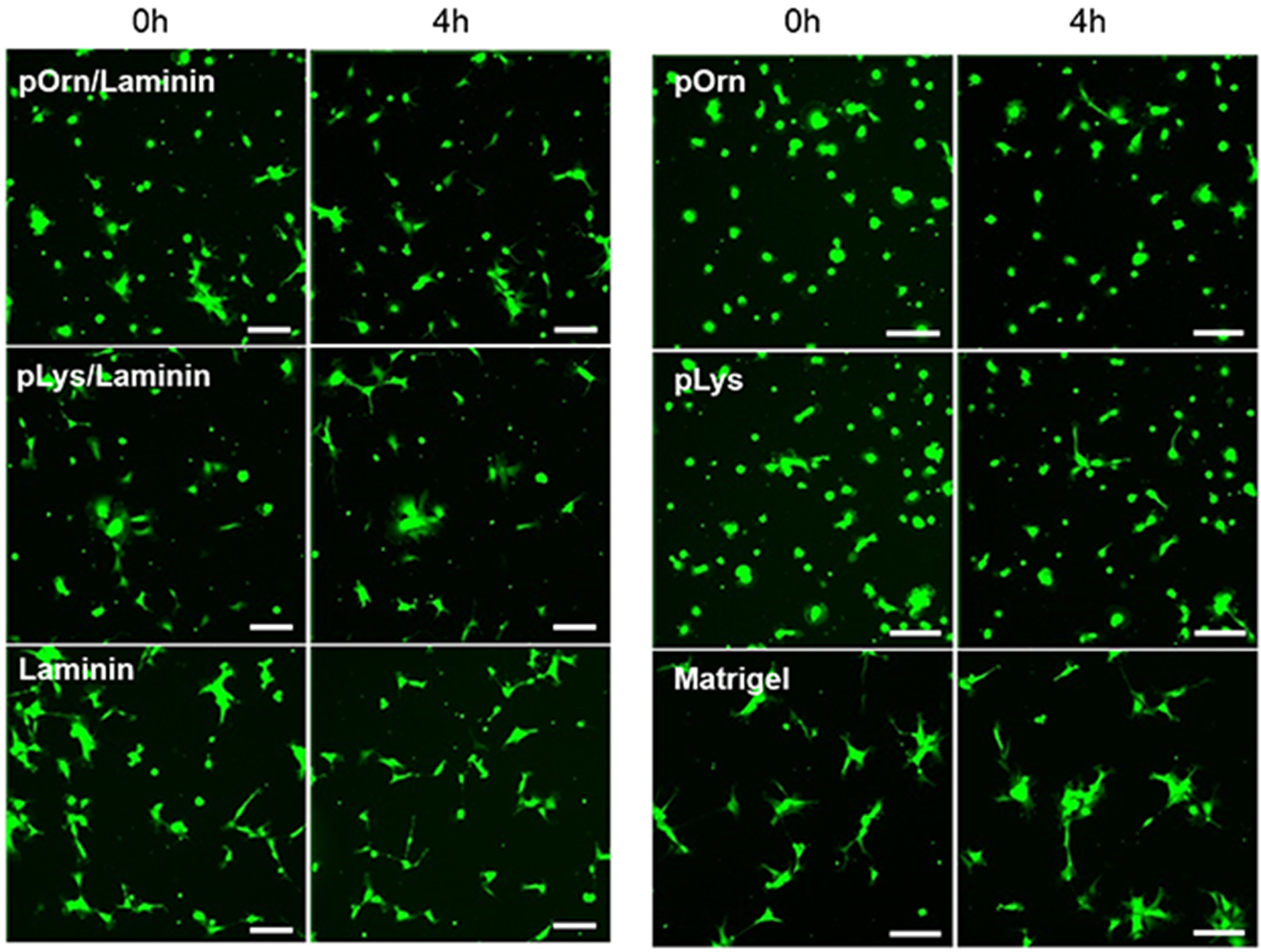

Supplement: Supplementary Figure 4 — Cell morphology of NPCs grown on different extracellular matrices. eGFP-expressing NPCs were seeded onto various extracellular matrices as indicated (pOrn, poly-ornithine; pLys, poly-lysine). Images were acquired before and 4 h after the addition of para-nitroblebbistatin (10 μM). Scale bars indicate 10 μm. [file Image_4.TIF]
